# Association between second-generation antipsychotics and newly diagnosed treated diabetes mellitus: does the effect differ by dose?

**DOI:** 10.1186/1471-244X-11-197

**Published:** 2011-12-15

**Authors:** Marianne Ulcickas Yood, Gerald N DeLorenze, Charles P Quesenberry, Susan A Oliveria, Ai-Lin Tsai, Edward Kim, Mark J Cziraky, Robert D McQuade, John W Newcomer, Gilbert J L'Italien

**Affiliations:** 1 EpiSource, LLC, Newton, MA, USA; 2 School of Public Health, Boston University, Boston, MA, USA; 3 Division of Research, Kaiser Permanente, Oakland, CA, USA; 4 Health Economics and Outcomes Research, Novartis Pharmaceuticals Corporation, East Hanover, NJ, USA; 5 HealthCore, Wilmington, DE, USA; 6 Otsuka Pharmaceutical Development and Commercialization Inc., Princeton, NJ, USA; 7 Department of Psychiatry, Washington University School of Medicine, St. Louis, MO, USA; 8 Global Health Outcomes Research, Bristol-Myers Squibb, Plainsboro, NJ, USA; 9 School of Medicine, Yale University, New Haven, CT, USA

## Abstract

**Background:**

The benefits of some second-generation antipsychotics (SGAs) must be weighed against the increased risk for diabetes mellitus. This study examines whether the association between SGAs and diabetes differs by dose.

**Methods:**

Patients were ≥18 years of age from three US healthcare systems and exposed to an SGA for ≥45 days between November 1, 2002 and March 31, 2005. Patients had no evidence of diabetes before index date and no previous antipsychotic prescription filled within 3 months before index date.

49,946 patients were exposed to SGAs during the study period. Person-time exposed to antipsychotic dose (categorized by tertiles for each drug) was calculated. Newly treated diabetes was identified using pharmacy data to determine patients exposed to anti-diabetic therapies. Adjusted hazard ratios for diabetes across dose tertiles of SGA were calculated using the lowest dose tertile as reference.

**Results:**

Olanzapine exhibited a dose-dependent relationship for risk for diabetes, with elevated and progressive risk across intermediate (diabetes rate per 100 person-years = 1.9; adjusted Hazard Ratio (HR), 1.7, 95% confidence interval (CI), 1.0-3.1) and top tertile doses (diabetes rate per 100 person-years = 2.7; adjusted HR, 2.5, 95% CI, 1.4-4.5). Quetiapine and risperidone exhibited elevated risk at top dose tertile with no evidence of increased risk at intermediate dose tertile. Unlike olanzapine, quetiapine, and risperidone, neither aripiprazole nor ziprasidone were associated with risk of diabetes at any dose tertile.

**Conclusions:**

In this large multi-site epidemiologic study, within each drug-specific stratum, the risk of diabetes for persons exposed to olanzapine, risperidone, and quetiapine was dose-dependent and elevated at therapeutic doses. In contrast, in aripiprazole-specific and ziprasidone-specific stratum, these newer agents were not associated with an increased risk of diabetes and dose-dependent relationships were not apparent. Although, these estimates should be interpreted with caution as they are imprecise due to small numbers.

## Background

Atypical, or second-generation antipsychotics (SGAs) represent an important advancement in the treatment of psychiatric symptoms and may have fewer extrapyramidal side effects than older antipsychotics [1]. However, there is a growing body of literature concerning the association between certain SGAs and risk of type 2 diabetes mellitus [2-9]. Studies regarding the effect of individual SGAs on diabetes vary in their conclusions, case definitions, and analytic approaches [10].

In a large study comparing the rate of treated diabetes in patients exposed to individual antipsychotics, we found that different SGAs were associated with varying levels of diabetes risk, ranging from no detectable increased risk (aripiprazole, risperidone, quetiapine and ziprasidone) to significant increases in risk (olanzapine and clozapine) [8]. This study was the largest post-marketing study to include newer atypical agents, aripiprazole and ziprasidone. Our findings were consistent with the American Diabetes Association consensus conference on this topic which found no association between the newer SGAs, ziprasidone and aripiprazole, and diabetes risk [2]. A recent study in Denmark conducted by Nielsen et al found a reduced risk of diabetes among aripiprazole users [2,3]. In addition, a study by Kessing et al found the risk of diabetes increased with first- and second-generation anti-psychotic polypharmacy [11]. However, the effect of SGA dose on diabetes risk was not examined directly in these earlier studies.

The purpose of this study was to examine whether any association between SGAs (aripiprazole, clozapine, olanzapine, risperidone, quetiapine, ziprasidone) and diabetes mellitus differs according to dose.

## Methods

The study population was formed using administrative and healthcare claims data from three United States (US) sites: (1) Kaiser Permanente Health Plan (Kaiser Permanente) (Northern California; 2.9 million covered lives during the study period); (2) HealthCore Integrated Research Network (HealthCore) (14.5 million covered lives in health plans across the US) and (3) PharMetrics (43 million covered lives from 73 health plans across the US). These sites were chosen because they maintain extensive electronic databases, including outpatient pharmacy, and inpatient and outpatient encounter and claims records. Outpatient pharmacy data include date of prescription fill, drug name, dose per pill, number of pills dispensed, and days-supply. While the electronic format of data from each site chosen is not identical, the principal investigators of this study followed a common protocol for collecting study data, have extensive experience compiling data from these multiple sources, have developed the site-specific algorithms needed to construct a single, uniform analytic dataset, and have established data management and quality control checks. A limited data set (defined by the Health Insurance Portability and Accountability Act (HIPAA) of 1996) was used and all researchers had HIPAA-required business associate and data use agreements in place before conducting the research. This study was approved by the Kaiser Permanente Northern California Institutional Review Board (IRB) and deemed IRB exempt at HealthCore and PharMetrics.

### Study Population

Patients ≥ 18 years of age, newly exposed to SGAs between November 1, 2002 and March 31, 2005 were included in the cohort. This inception cohort consisted of all patients exposed to an SGA for at least 45 days and continuously enrolled for at least 3 months before and 6 months after the date of first prescription (index date) with no evidence of diabetes using all available historical data prior to the index date and no previous antipsychotic prescription (first generation or SGA) filled within 3 months of the index date.

### Exclusion of Patients with Previous History of Diabetes Mellitus

Previous history of diabetes used all available historic data and was defined using the following criteria: (1) at least one emergency department visit with an International Classification of Diseases, 9th Revision (ICD-9) coded diagnosis indicative of diabetes (250.xx); or (2) at least two outpatient visits coded with diabetes (250.xx); or (3) at least one inpatient stay with an ICD-9 coded diagnosis indicative of diabetes (250.xx); or (4) filled at least one prescription for an oral anti-diabetic agent or insulin. Diagnosis codes or medication utilization were used as exclusion criteria to provide a conservative approach to excluding patients with a previous history of diabetes.

### Classification of Antipsychotic Exposure

Data analysis accounted for drug switching and non-consistent use of drug treatment by categorizing person-time exposed to individual antipsychotic agents. Patients contributed exposed person-time to individual antipsychotics beginning 45 days after index date, continuing for the duration of the days-supply for an individual prescription. Person-time accumulated, as described previously, until the first of the following occurred: (1) patient switched to a different antipsychotic; (2) prescription filled for anti-diabetic pharmacotherapy; (3) disenrollment from health plan; (4) death; (5) end of study period. Exposed person-time categories were formed for aripiprazole, olanzapine, risperidone, ziprasidone, and quetiapine. Clozapine was not included due to insufficient numbers.

Daily dose prescribed for each agent was calculated by multiplying number of pills dispensed by dose per pill, divided by days supply for that prescription episode. Blinded to study outcome and exposure, the following decision rules were applied to tabulate dosing episodes to determine whether decision-rule modification was necessary:

1. If patient received consecutive prescriptions for the same dose of medication, but the prescription was filled within (+/-) 14 days of the claims start/end date, the gaps were bridged and patient was classified as continuously exposed to the prescribed/recorded dose.

2. If patient filled 2 or more prescriptions on the same day, with the same days-supply and same dose, the days-supplies were summed.

3. If patient filled 2 or more prescriptions on the same day, with the same dose but different days-supply recorded, the days-supplies were summed.

4. If patient filled 2 or more prescriptions on the same day, with different doses:

a. If the days-supply between prescriptions was within (+/-) 14 days, daily dose was summed to the maximum days-supply between the 2 prescriptions.

b. If the days-supply between prescriptions was greater than 14 days, dose was considered switched to the second prescription strength (after 14 days).

c. If the days-supplies were equal, daily dose was summed.

d. If the days-supplies were equal and there were ≥2 records on the same day (e.g. record one--100 mg/30 days, 200 mg/30 days; record two--100 mg/30 days, 200 mg/30 days), we summed the daily dose and divided by the number of records and summed the days-supply (e.g. 300 mg/60 days).

Using these decision rules, we obtained (blinded) clinical input to determine whether data/decision rules reflected expected clinical dosing distributions. The dosing distributions for each drug were reviewed by a team of clinical experts consisting of two clinicians (one clinician was the study sponsor lead; the second clinician was an outside academic consultant), and three PhD-level epidemiologists (two epidemiologists were from the contract research organization and the third represented the study sponsor). To determine dosing distributions, each expert team member was provided a distribution of the dosing for each drug in the study. Blinded to study outcome, the team collectively reviewed the distribution for each drug and determined--based on clinical and statistical judgment--that tertile categorization was appropriate because the distribution of outcomes was too sparse to allow more granular analyses. The team addressed and deleted extreme outliers in dosing (e.g., clinically implausible doses indicative of data entry errors).

### Study Outcome (Newly Treated Diabetes)

Patients exposed to SGAs for at least 45 days during the period January 1, 2002 through March 31, 2005 were identified and followed for the outcome of treated diabetes. To ensure ample exposure for evaluation, individuals were followed from the 45^th ^day after index date through the earliest of the following events: (1) prescription filled for anti-diabetic pharmacotherapy; (2) disenrollment from database/health plan; (3) death; (4) end of study period.

### Statistical Methods

Current daily antipsychotic dose (categorized by tertiles for each drug; quantified in person-time) was calculated as a time-dependant covariate (i.e. dose could change over follow-up time). Newly treated diabetes mellitus was identified using pharmacy data to determine patients exposed to anti-diabetic therapies. Hazard ratios (HRs) for diabetes across dose tertiles for each SGA were calculated via Cox proportional hazards regression using the lowest dose tertile as the reference, and adjusting for age, sex, study site, year of cohort entry, history of antipsychotic use (>3 months prior to index), exposure to other pharmacotherapy (alpha blockers, beta blockers, statins, corticosteroids, fibrates, lithium, oral contraceptives). We fit separate models for each SGA. Tests for trend across dose within each SGA were performed by coding increasing dose as 1, 2, and 3 and treating as a continuous variable in the Cox regression model.

## Results

The mean age of patients was 44 years, and 40 percent were male; exposure to beta blockers, systemic corticosteroids, and valproate were most common, and 5% of patients were obese according to ICD-9 codes (Table 1).

**Table 1:** Demographic and Clinical Characteristics of Patients Exposed to Atypical Antipsychotics, November 2002-March 2005 (N = 49,946)

Characteristic	N (%)
**Sex**	
Male	19,981 (40.0)
**Age (mean (s.d.^a^))**	43.9 (19.2)
**Index Year**	
2002	5,407 (10.8)
2003	24,438 (48.9)
2004	18,186 (36.4)
2005	1,914 (3.8)
**Past use of antipsychotics**	
Aripiprazole	1,966 (3.9)
Clozapine	60 (0.1)
Olanzapine	13,826 (27.7)
Quetiapine	10,632 (21.3)
Risperidone	11,367 (22.8)
Ziprasidone	1,367 (2.7)
First generation antipsychotics (typicals)	1,714 (3.4)
**Pharmacotherapy exposure **^b^	
Alpha blockers	1,975 (4.0)
Beta blockers	9,479 (19.0)
Systemic corticosteroids	12,254 (24.5)
Fibrates	960 (1.9)
Lithium	6,662 (13.3)
Nogesterol oral contraceptives	487 (1.0)
Statins	5,171 (10.4)
Thiazide diuretics	7,561 (15.1)
Thiazide-related diuretics	219 (0.4)
Valproate	10,314 (20.7)
Hydantoin anticonvulsants	1,020 (2.0)
**ICD-9 coded comorbidity**	
Obese	2531 (5.1)

^a ^s.d. = standard deviation

^b ^variables included in parent study^8^

The rate and hazard ratios of treated diabetes in patients exposed to SGAs by dose and drug are reported in Table 2. Olanzapine exhibited a dose-dependent association with rate of diabetes (compared to the lowest tertile (<5 mg)): HR = 2.5 for the highest tertile (≥ 10 mg) (95% CI 1.4, 4.5); and HR = 1.7 for the intermediate tertile (5-<10 mg) (95% CI 1.0, 3.1). Quetiapine and risperidone exhibited elevated rates at the top tertile doses when compared to the lowest tertile: quetiapine (>150 mg vs. ≤ 50 mg) HR = 2.5 (95% CI 1.3, 4.7); risperidone (≥ 2 mg vs. < 1 mg) HR = 2.1 (95% CI 1.0, 4.4). No evidence of increased rate of diabetes was observed for quetiapine and risperidone at the intermediate dose tertile. Although the estimates are imprecise for the newer SGAs aripiprazole and ziprasidone, there was no evidence of dose dependence or elevated rate of new onset diabetes in these agents.

**Table 2:** Rate and Hazard Ratios of Treated Diabetes in Patients Exposed to Second-Generation Antipsychotics^a^, By Dose^b ^and Drug

Dose category	Patients exposed (N)	Events (N)	Person-years	Diabetes rate (per 100 person-years)	**Adjusted**^c ^**HR**^d^ **(95% CI**^e^)
**Aripiprazole**					p^f ^= 0.43
≥15 mg	1321	4	371	1.1	1.3 (0.1, 12.2)
10 - <15 mg	988	1	214	0.5	0.6 (0.04, 9.8)
<10 mg	788	1	145	0.7	Reference

**Olanzapine**					p^f ^= 0.002
≥10 mg	5921	58	2176	2.7	2.5 (1.4, 4.5)
5 - <10 mg	6761	41	2118	1.9	1.7 (1.0, 3.1)
<5 mg	4398	15	1361	1.1	Reference

**Quetiapine**					p^f ^= 0.007
>150 mg	4686	34	1686	2.0	2.5 (1.3, 4.7)
51 -150 mg	5525	15	1610	0.9	1.2 (0.6, 2.5)
≤50 mg	6516	13	1838	0.7	Reference

**Risperidone**					p^f ^= 0.10
≥ 2 mg	5103	23	1852	1.2	2.1 (1.0, 4.4)
1 - <2 mg	5187	15	1649	0.9	1.4 (0.6, 3.1)
<1 mg	4353	10	1372	0.7	Reference

**Ziprasidone**					p^f ^= 0.60
>80 mg	624	1	186	0.5	0.3 (0.03, 3.4)
41 - 80 mg	671	2	170	1.2	0.6 (0.1, 4.0)
≤40 mg	725	3	181	1.7	Reference

^a^Results from clozapine not included because numbers were insufficient.

^b^Dose per day

^c^Adjusted for age, sex, study site, year of cohort entry, history of antipsychotic use (>3 months prior to index), exposure to other pharmacotherapy (alpha blockers, beta blockers, statins, corticosteroids, fibrates, lithium, oral contraceptives, thiazide (and related) diuretics, valproate, hydantoin), and obesity

^d^HR = hazard ratio

^e^CI = confidence interval

^f ^test for trend

## Discussion

In this large, multi-site epidemiologic study, the risk of diabetes for persons exposed to olanzapine, quetiapine, and risperidone appears to be dose-dependent. Quetiapine and risperidone exhibited elevated risk at top tertile doses, but no evidence of increased risk at the intermediate dose tertile. Consistent with dosing guidelines and the literature, the top tertile doses for quetiapine and risperidone are indicated for treatment of schizophrenia, bipolar disorder and depression [2,3,12-14]. Lower doses may be more consistent with off-label uses (i.e. sedation, irritability) [14,15]. Unlike olanzapine, quetiapine, and risperidone, aripiprazole and ziprasidone did not exhibit a dose dependent association with diabetes. Of note, the baseline (reference) diabetes rate was similar for all study SGAs except ziprasidone (where numbers were limited).

This study is subject to several limitations. Given sample size limitations for the newer agents, aripiprazole and ziprasidone, precision in hazard ratio estimation is low. As more individuals are exposed to these agents over time, more precise estimates can be made. However, our findings are consistent with a recent study by Guo which utilized different dosing cut points in a different population and found similar estimates for these newer agents [16]. While it is likely that patients with more severe psychiatric disease receive higher doses of SGAs and this could potentially lead to confounding by indication, the impact may be minimized through normalization of the dosing across medications using clinically significant tertile cut points. This study is subject to the limitations inherent in using administrative databases. Prescribed daily dose was not available; instead, daily dose was calculated using an approach commonly utilized in analyses of dose from administrative databases. Due to data availability and quality, this study does not include inpatient prescriptions. As a result, hospitalized patients receiving specific SGAs were not included. The incidence of diabetes may be underestimated, as a result. However, the relative effect estimates should be the same if hospitalization does not differ by medication. Given that the inception cohort was formed using a 3-month window of no evidence of previous antipsychotic exposure, it is possible that patients with previous antipsychotic exposure were included. In addition, we were unable to evaluate polypharmacy, a factor shown by Kessing et al to be associated with diabetes risk [11]. If exposure to polypharmacy is more prevalent in patients exposed to the newer agents (i.e. aripiprazole or ziprasidone), this may bias the results. This study is also subject to limitations inherent in observational studies including confounding, for example between higher doses and more severe illness where medical co-morbidity may be more common. However, given that analyses included internal comparisons (i.e., within-drug comparisons), confounding is likely minimal.

## Conclusions

This study suggests a dose-response relationship between certain SGAs and risk of diabetes mellitus. However, the number of patients exposed to the newer antipsychotics (aripiprazole and ziprasidone) is limited, resulting in reduced precision in hazard ratio estimates. Future goals include updating this sample to obtain greater precision around the estimates for the two newer agents, aripiprazole and ziprasidone.

## Competing interests

Drs. Ulcickas Yood, DeLorenze, Quesenberry, Oliveria, Cziraky, and Newcomer and Ms. Tsai declare that they have no competing interests.

Edward Kim was an employee of Bristol-Myers Squibb at the time this study was executed and holds shares in Bristol-Myers Squibb, manufacturer of aripiprazole.

Robert McQuade is an employee of Otsuka Pharmaceutical Development and Commercialization Inc. and is a former employee and shareholder in Bristol-Myers Squibb, manufacturer of aripiprazole.

Gilbert L'Italien is an employee and shareholder in Bristol-Myers Squibb, manufacturer of aripiprazole.

John W. Newcomer, M.D., over the past three years, has received research grant support from The National Institute of Mental Health (NIMH), NARSAD, Sidney R. Baer Jr. Foundation, Bristol-Myers Squibb, Pfizer, Inc. and Wyeth; he as served as a consultant to AstraZeneca Pharmaceuticals, Bristol-Myers Squibb, BioVail, H. Lundbeck, Janssen Pharmaceutica, Obecure, Otsuka Pharmaceuticals, Pfizer, Inc., Sepracor, Inc., Solvay Pharma, Inc., Vanda Pharmaceutica and Wyeth Pharmaceuticals; he as been a consultant to litigation; he has been a member of Data Safety Monitoring Boards for Dainippon Sumitomo Pharma America, Inc., Organon Pharmaceuticals USA Inc., Schering-Plough/Merck and Vivus, Inc; finally he has received royalties from Compact Clinicals/Jones and Bartlett Publishing for a metabolic screening form.

## Authors' contributions

All authors participated in writing and editing the manuscript. All authors read and approved the final manuscript.

In addition, each author made the following specific contributions:

MUY participated in the study design, data acquisition, consultation on statistical analysis, and interpretation of results. GND participated in the study design, acquisition of data, oversight of statistical analyses, and interpretation of results. CPQ participated in the study design, acquisition of data, conduct of statistical analyses, oversight of statistical analyses, and interpretation of results. SAO participated in the study design, data acquisition, and interpretation of results. AT participated in the acquisition of data, conduct of statistical analyses, and interpretation of study results. EK participated in study conception and interpretation of results. MJC participated in study design, data acquisition, and interpretation of study results. RDM participated in study conception and interpretation of results. JWN participated in study conception, design, and interpretation of results. GJL participated in study conception, design, and interpretation of results.

## Pre-publication history

The pre-publication history for this paper can be accessed here:

http://www.biomedcentral.com/1471-244X/11/197/prepub
